# How school nurses experience and understand everyday pain among adolescents

**DOI:** 10.1186/s12912-017-0247-x

**Published:** 2017-09-13

**Authors:** Magnhild Høie, Kristin Haraldstad, Gudrun Rohde, Liv Fegran, Thomas Westergren, Sølvi Helseth, Åshild Slettebø, Berit Johannessen

**Affiliations:** 1 Faculty of Health and Sport Sciences, University of Agder, Kristiansand, Norway; 2 Faculty of Health Sciences, Oslo and Akershus University College of Applied Sciences, Oslo, Norway

**Keywords:** Pain, Biopsychosocial understanding, Adolescents, School nurses

## Abstract

**Background:**

Pain problems are a rapidly growing health problem found among both children and adolescent, and about 15–30% have reported chronic pain problems. School nurses in Norway meet adolescents with various ailments, including pain. Yet research on how school nurses perceive the pain experienced by adolescents is limited. The aim of the present study was to explore how school nurses explain and experience the everyday pain of adolescents.

**Method:**

A qualitative study with an explorative design comprising five focus group interviews. Each group consisted of three to five school nurses. Seventeen female school nurses in five junior high schools in Norway, age range 29–65 years participated. To cover the issues a semi structured interview guide was used. The transcribed text was analysed with qualitative content analysis.

**Results:**

The experience of school nurses with adolescents’ pain in everyday life is mainly that pain is a social, physical, and psychological phenomenon. School nurses experienced that everyday pain is reflecting: 1) high expectations, 2) difficult relationships and traumatic experiences and 3) an unhealthy lifestyle. School nurses have ambivalent attitudes to medicalisation of pain.

**Conclusion:**

Despite of a biopsychosocial understanding of pain, the school nurses maintained referral practice of medical examinations, with the results that many adolescents became shuttlecocks in the health system. Although the school nurses´ were sceptical of the tendency towards medicalization in society, it appears that they actually help maintain this tendency.

## Background

Pain problems are a rapidly growing health problem among both children and adolescents and are recognized as a significant public health challenge [1–5]. Disturbingly, the literature shows that as many as 15–30% of adolescents suffer from persistent or chronic pain conditions [6, 7]. The prevalence varies between studies because of the use of different methods, age groups, and use of questionnaires, however several studies have shown a trend toward a high prevalence of pain in children and adolescents, and that this is an increasing problem [8, 9]. Such pain has a major impact on key aspects of daily life, including sleep patterns, school functioning and school absenteeism, loss of appetite, not meeting friends, and the inability to participate in sports [1, 10–12]. Pain often relates to stressful conditions in the everyday lives of adolescents, with some relying heavily on analgesics to cope [8, 9].

Pain is a complex psychosomatic experience, and as stated, often relates to different kinds of stressors, including diminished physical activity, sleep problems, poor eating habits, bullying, harassment, schoolwork pressure, and poor treatment by teachers and peers [13, 14]. The evidence also suggests that children from families with low socioeconomic status suffer more commonly from pain [15] and that parental relations play an important role in the self-reported health of adolescents [16], such that the parental response (modelling, reinforcement of vulnerability) to pain will influence how adolescents experience pain [17]. Study shows that much time spent with visual media can also be a risk factor for pain [18]. In the references we use there are no gender differences.

In the last decade, the main approach to pain in health care has been biopsychosocial [19, 20]. For example, a biopsychosocial approach could include the reciprocal influence or relations with: 1) stressful life events, 2) injury, trauma, or disease, 3) lifestyle, for example, physical inactivity and stress, and 4) psychological factors, for example, stress, family, relations, and friends. These factors further imply the self-perpetuation of pain, cycles of pain, retention, sleep problems, and anxiety or depression [19, 21]. In terms of work in this area, Kozlowska et al. [19] and Rohde et al. [21] found that teachers possess a biopsychosocial understanding and approach to the pain experienced by adolescents and that this understanding influences the role of teachers as significant others in the lives of adolescents in the school setting.

Along with parents and teachers, school nurses (SNs) meet children and adolescent regularly. In Norway’s school health service, it is a statutory municipal initiative that SNs are a key player. Every school must have a SN with specializing in public healthcare. Someone has a master degree.

Pupils should be able to visit the nurse whenever they feel the need, however, SNs have limited time and resources. Because they have a key role and a position that facilitates dialogue with the adolescent, teachers, and parents, we believe that their descriptions and perceptions regarding young people with pain make a valuable contribution to a greater understanding of young people’s everyday pain.

The importance of the relationship between SNs and adolescents with health complaints is highlighted by Pavletic [22] and Svebak et al. [23], who found that the level of student complaints tended to decline with access to the school nursing service. Further, Borup et al. [24] concluded that visiting the SN might reduce students’ proneness to the use of medication when they experience aches and psychological problems. However, concerning abdominal pain, Youssef et al. [25] found that SNs were unclear on the epidemiologic and etiologic features of recurrent abdominal pain, and that their negative views may inadvertently contribute to the anxiety felt by affected children.

Clearly, there is a gap in the literature regarding SN experiences of pain in adolescents. The aim of the present study is therefore to explore how SNs explain and experience the everyday pain of adolescents.

## Method

To gather information about experiences, we selected a qualitative design with roots in phenomenology and hermeneutics [26]. We collected data through focus group interviews with SNs. In focus groups, people with similar experiences gather together to discuss a given topic and conversation between the participants is central. The dynamics between the participants allows for insights of a different nature than those we ordinarily obtain through individual interviews [27]. This method also helps participants reflect upon their own practices [26]. The focus group is led by a moderator and a co-facilitator who facilitate an open atmosphere where participants feel that they can express personal and possibly conflicting views. Our focus groups included between three and five participants.

### Recruiting and sample

The criterion for inclusion was a minimum of two years’ experience as a SN for adolescents in a junior high school (age 13–16 years). To obtain maximum variation, we used purposive sampling according to districts with adolescents from multiple cultural and social demographic backgrounds and across urban and rural areas. We contacted school head nurses and they asked the school nurses to participate voluntarily. A total of 40 SNs was contacted, and 17 accepted the invitation. The participants were 29–65 years of age, all female, with 2–35 years of experience as SNs. The five focus groups included between three and five participants.

### Data collection

During the spring of 2013, we conducted five focus group interviews at each of the participant’s workplaces. Two researchers participated in the interviews, one as moderator and one as co-facilitator. The interviews were organized around a semi structured interview guide. The interviews focused on SNs’ perceptions of the causes of pain and the way adolescents expressed and managed every day pain. (Examples of key questions: “How do you experience pain among adolescents? What is your opinion about how adolescents cope with pain? What are your thoughts about the causes of pain among adolescents?”). The interviews lasted roughly 90 min and were audio-recorded and transcribed verbatim into 150 pages of material.

### Data analysis

We used qualitative content analysis inspired by a phenomenological hermeneutic approach in which the experiences of informants are at the centre and researcher preunderstandings are included in the interpretation of the findings. Two researchers (MH and BJ) in close collaboration analysed the interview text using an inductive approach that started with meaning condensation, categorization, and thematization. We followed the four analytic steps in Malterud [26]: 1) reading all the material to obtain an overall impression and noting preconceptions; 2) identifying units of meaning and coding different aspects of the participants’ experiences; 3) condensing and abstracting the meaning within each of the coded groups; and 4) summarizing the contents of each code group to generalize descriptions and concepts to a main theme reflecting the participants’ most important experiences.

**Fig. 1:**
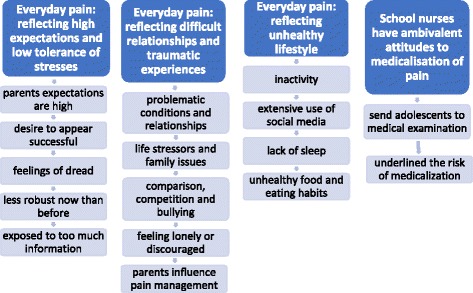
Key factors of every day pain

After reading through the data as a whole, the overall impression was that the SNs perceived pain in adolescence to be a complex phenomenon (step one). The second step was to code and sort the text into meaning units. During the third step, we gathered the text with the same content and condensed it into subthemes. Finally, we sorted the subthemes into four categories, see Fig. 1, page 7. We discussed the analysis until we reached a consensus among the research group consisting of the eight authors of this article. The researchers are all nurses with a health promotion perspective, two with a background in psychology (MH, KH), and two public health nurses (SH, BJ). The Focus groups were carried out in Norwegian. The quotations were translated by a professional translation agency.

### Ethical considerations

The ethical principles of autonomy, beneficence, non-maleficence, and justice were assured following the Helsinki Declaration. The study was approved by the Norwegian Social Science Data Services (NSD) (Approval No. 32829) for safe handling and storing of data. The participants were given written as well as oral information and written informed consent was obtained. Voluntary participation and confidentiality were assured during the collection, handling, and reporting of the data.

## Results

SNs emphasized that the adolescents’ everyday pain was complex, linked to psychosocial life situations where expectations, relationships, and lifestyle were key factors (Fig. 1). At the same time, they highlighted physical aspects of pain by sending adolescents for frequent medical examinations. Nevertheless, it was underlined that the adolescents pain was real for the adolescents themselves, and therefore had to be taken seriously. All SNs participated in the discussions and are represented in the quotations. The findings did not reveal any differences between SNs from rural or urban schools, regarding their experiences.

### Everyday pain: Reflecting high expectations and low tolerance of stresses

All SNs in this study associated adolescent pain with high expectations, both from the adolescents themselves and from their surroundings: “*To always be the best is demanding. A lot of young people experience the pressure of always being at their best.*” SNs gave multiple examples of how the parents of the adolescents expected high grades and put pressure on their children: “*A girl had a C average. And when she came home one day, her dad told her ‘this is not good enough, because I know that you can do better.’ This comment really bothered the girl, and she thought it was very painful. So, she worked hard. But she felt ‘I can’t do it, I’ll never get it.’ I also have a boy coming to my office, he wants to become a doctor. But his grades are not good enough, and because of that he has problems sleeping. So, his parents expect him to become something great, right?*”

Much was also said about self-imposed stress and all the “good girls.” One SN reported: “*Some of them are very hard on themselves. They have very high demands, they can have a B, right, but that’s not good enough.*” It was also suggested that some had too much responsibility, and a high degree of loyalty to home. This was related to different types of bodily ailments among some pupils.

SNs explained how many adolescents are in pain when they dread certain events, particularly when they have to be in the spotlight, such as making a presentation to others or meeting with certain/particular teachers. Such feelings of dread can be associated with headache, stomach pain, sleep problems, and absence from school. One of the SNs commented: “*To dread something in this way can be felt in different/various places in the body, maybe it’s in the stomach. Some dread the breaks, others dread being alone, there are the ones who dread certain comments, and finally, those who dread physical training*.”

Several of the SNs emphasized that adolescents are less robust now than before, that there was much absence and more complaints: “*Most of us will have hardship in life, and then I think many have a tendency always to think that some professional is going to help them. But they need to learn how to deal with some adversity.*” They meant that many young people have expectations of “a pain-free life” with the immediate need to satisfy their needs. One said: “*I believe an individual’s needs are much stronger now than earlier. They are less robust to life itself. I wish they were more robust, because there will be challenges in life.*” All SNs experienced that adolescents extensively used painkillers such as paracetamol: “*Some adolescents have paracetamol in their pencil boxes, and many of them get the pills from their mothers.*”

Another SN stressed the lack of demands and responsibility: “*I think they are being guided for too long… somebody call it a curling generation, they are less independent and they do not take any responsibility.*” Some SNs compared the lives of adolescents today with their own 20–30 years earlier, and agreed that the adolescents of today are exposed to too much information, conveyed too much information and had too little responsibility. They connected this to different types of pain.

### Everyday pain: Reflecting difficult relationships and traumatic experiences

SNs conveyed a clear perception that pain was connected to problematic conditions and relationships: “*The adolescents start talking about where they have pains, but there is often something else behind it. After a while, it becomes evident that the physical pain is not the real issue. They have a stomach ache or a headache, but the real problem is perhaps something social, something at home, something with their well-being or other things.*” The conversation with the adolescent could start with the physical pain, but often ended up with a conversation about life stressors.

The SNs claimed that young people adapt to family issues and problems, and that this became a psychological problem that attached to the body and created different physical ailments: “*I think adolescents adapt to an unhealthy family interacting. I think this often, very often. I then think that the pain they are carrying is due to arguments in the home; they don’t get on well with their mom or their dad, for example, because the level of conflict is very high.”*

The SNs emphasized that many adolescents had a desire to appear successful and have many friends, to be popular and cool. Facebook and blogs were both denoted as “creating contacts,” “far from reality,” and a platform/arena for comparison, competition, and bullying. The SNs meant that it was very important for the adolescents to be available online. SNs also said that adolescents compared themselves with others and could feel unsuccessful or left out, giving them mental pain in terms of loneliness and depression. One of the SN told: “*Sometimes, with those I know better… I understand that they have extremely high demands on themselves ...or from their parents…first, they talk about their daily physical pain. Sometimes, someone also touch upon issues such as being sad, anxious and lonely.”*

The SNs said that it was completely normal among the adolescents to rank one another according to looks, “prettiness,” “slimness,” and other external characteristics, and they agreed that much revolves around looks and popularity. It was said that “*then there are those who never get on these lists. It could be our age bullying”.* “*It could be our generation’s way of saying/stating that you are not one of us.*” The SNs also connected pain to the fact that the adolescents felt unsuccessful, on the outside, lonely, or discouraged.

The SNs often talked about the fact that pain could be related to traumatic events such as violence, divorce, or death in the adolescent’s close family. One SN talked about a girl who had a stomach ache for three years, even though she was examined at the hospital several times without result. No one linked her pain to having lost a close family member. However, when she finally joined a grief group, the stomach ache disappeared. Another SN talked about a girl who came to her because she was experiencing a great deal of pain, in her stomach, head, and legs. After having spent some time with her, she revealed that her brother was in prison. The girl thought about this constantly, but dared not tell anyone.

Some stressed that there were taboos in the adolescents’ families, such as things they would not speak of, for example, if the mother or father were alcohol or drug abusers, and they often tried to protect their parents by covering for difficult family conditions. Several of the SNs provided examples of adolescents who became “caregivers” for their parents: “*After finally getting her to speak, she revealed that she had to do most of the housework, she had to support and help her parents, younger brothers and sisters… probably she was very tired of keeping it all a secret. She also told me she was so ashamed of her home situation... no wonder that she had bodily pain… children and adults are so loyal.*”

All SNs emphasized that they particularly worked with problems concerning bullying. They had a so-called zero tolerance policy toward bullying, but at the same time admitted that it was not possible to uncover everything. During the discussions, dissatisfaction at school was often cited as a cause of pain, for example, bullying, a lack of friends, being afraid of certain teachers, and so on. One of the SNs commented: “*If their head hurts and they are unable to go to school, it is natural to try to identify the problems.”*

They also postulated that adolescent pain was related to the way parents (especially mothers) dealt with pain. Mothers who took painkillers frequently offered them to their children.

### Everyday pain: Reflecting an unhealthy lifestyle

The SNs also talked about adolescent inactivity and extensive use of social media and connected this to the fact that many were bothered with headache, neck, back, and shoulder pain. Moreover, they suggested that young people could not put their cell phones or computers away, causing them to go to bed very late. The result was that a lack of sleep made it hard to get up in the morning, they were tired, and headaches increased: *“Many sleep too little*”, one of the SN commented “*thus they experience poor quality of sleep and then their head hurts, but also their neck and back; a lot of muscle aches.*” In addition to spending too much time on social media, many adolescents were physically inactive and many took advantage of their “pains” to skip gym classes*.*

Food and eating were also a theme that the SNs connected to pain problems. They particularly emphasized how young girls with headaches and stomach-aches often admitted that they ate and drank inadequately, in terms of both quantity and frequency. They could go without breakfast and lunch, and only have a small portion of dinner, without being able to see the connection between their daily food intake and their bodily pains. The SNs did not talk about those that were overweight.

### SNs have ambivalent attitudes to medicalisation of pain

Even if the SNs strongly underlined the complexity of the pain, they talked much about their responsibility to send the adolescents to medical examination at doctors and hospitals: “*It is important not to miss a serious diagnosis*” one of them concluded*.* “*To be on the safe side*,” another said. In particular, adolescents with repeated abdominal pain were often referred for somatic examination, often under pressure from their parents. SNs admitted that most of them came back from examinations with the same pain, and without any somatic diagnosis. Nevertheless, all SNs underlined the risk of medicalization, stating that the young people of today were more fragile and were seen to run to the health staff for relatively minor complaints: “*The adolescents with everyday pain often become shuttlecocks in the system. This is not good. Their* *real* *challenge often is that they return with* *unchanged* *health problems*. W*ithout a change in their social situation, their deficient coping, negative stress, or their personal strength, the everyday pains keep on.*” In addition to consistency in referral practice, SNs also considered themselves as individuals the adolescents had confidence in, and they had positive experiences of their dialogues with the adolescents: “*I am not too close and not too far*,” one of them said. “*This position creates confidence. The adolescents may tell me about themselves, without being afraid of being disgraced.*” The SNs looked upon themselves as both professionals and as fellow human beings.

## Discussion

The aim of the present study was to explore how SNs explain and experience the everyday pain of adolescents. Our findings show that when young people’s every day pain had obvious physical reasons, SN underlined that referring to medical evaluation or treatment was their obvious duty, as it is not a nurse task to investigate medical conditions. The challenge, as the nurses experienced, was that in the absence of clear physical causes, adolescents everyday pain may be symptoms of underlying causes, and just as often could be justified in psychological or social conditions. According to the SNs, the great challenge was not to contribute to medicalization of adolescents’ pain and, at the same time, nor to miss medical conditions. Above all, they underlined that pain was real for the adolescents and had to be taken seriously, either the pain clearly had a physiological cause, or it was understood as linked to psychosocial conditions. In that context SNs were concerned about what was behind the pain. In the situation of no obvious physiological explanation for pain, the SNs mainly associated it with the adolescents themselves or significant others having expectations exceeding their coping skills, especially concerning school performance. SNs also interpreted that adolescent pain could result as a consequence of difficult relations and traumatic incidents relating to significant others, and argued that adolescent lifestyle itself might result in recurrent pain.

Adolescents’ experiences of never being good enough were emphasized by the SNs. Lask [28] has shown how well-behaved and apparently well-adjusted children may develop long-lasting and functional restraining conditions. When high family standards of performance—“*always to do one’s utmost*”—had been integrated as a personal standard, this resulted in sustaining stress and anxiety manifesting itself into bodily pain [28].

The SNs described adolecents` as carriers of their parents’ concerns, and studies have highlighted the importance of SNs paying special attention to parent–adolescent relations in their work with adolescents in pain [16]. Garralda [29] undertook a systematic review to map different psychosocial factors that seemed particularly correlated with long-term pain among children, and found a high incidence of anxiety in connection with family health problems [29]. Poutiainen et al. [30] also highlighted the importance of taking into account family characteristics in health care contacts, and the targeting of preventive health care measures at the entire family.

Research supports the notion that psychological strain and stress can cause pain and somatization of symptoms [13, 14, 31]. On occasions, the SNs were given an insight into complicated home situations closely associated with shame. Examples of how some adolescents made every effort to conceal difficult conditions at home were highlighted in the focus groups (such as the girl who was ashamed of her brother being in prison). Stern [32] describes well how feelings of shame and guilt can be expressed as pain.

Walter [33] found that lifestyle behaviours such as skipping meals, water intake, tobacco and alcohol use, and physical inactivity predicted recurrent headache. The SNs in this study found that adolescent inactivity and extensive use of social media were related to headaches, neck, back, and shoulder pain, as well as sleep problems, all of which are confirmed by previous research [1, 10–12].

It was important for SNs to eliminate physical causes of adolescent pain, although they found that physical causes were extremely rare. SNs experienced pain as mainly caused by psychosocial factors. Despite this acknowledgement, they emphasized the importance of referring adolescents to repeat somatic examinations. Previous studies indicate that unclear states of condition may increase the tendency to further extensive explorations (e.g. Holtedahl) [34]. Their practices reflect a dualistic comprehension that the pain condition can be explained either physically or psychosocially [35]. This may relate to the fact that many—and not least parents—find it easier to accept physical ailments, and attach stigma to psychosocial conditions [36]. It appears that the SNs become partners in such an attitude. The group discussions frequently revealed a certain amount of scepticism toward the increasing medicalization of society, without connecting it to their own practice [37]. This seems paradoxical, not least because of their own experiences of these adolescents as shuttlecocks in the somatic health service. It may seem as if they maintain the adolescents’ shuttle in the system and contribute little to solve their possible underlying causes of pain. For the most part, the education of SNs is in a somatic tradition, and as nurses, they have worked closely with medical doctors. This could contribute to explaining their tendency to book medical appointments.

SNs had the impression that adolescents used analgesics extensively when experiencing pain, as was also found in other studies [8, 9]. Furthermore, the SNs described the adolescents as rather fragile and expressed a clearly sceptical attitude toward their use of analgesics. They wanted to create a counterculture, one that would accept the normality of having pain from time to time, but not wanting to pathologize every discomfort. This also reflects a medicalization paradox in that at the same time SNs continued to refer adolescents for medical examinations.

An important aspect of the psychosocial causes of pain is linked to being excluded from the digital community including peers, in addition to a devaluation of themselves concerning a lack of popularity and position in social media. Adolescents today spend much of their time on social media, and confirmed that “likes” from others might be of great importance [38]. Being subject to social exclusion and negative comments over time could harm self-esteem, contributing to psychical nuisance among adolescents [39]. The SNs also emphasized the tendency of adolescents to compare themselves with each other on social media. Festinger’s theory of social comparison draws on the fact that we form interpretations about ourselves by comparing our abilities/capabilities and skills with others, especially those who resemble us [40]. If the comparisons result in experiencing oneself as less successful than others, it may also result in depression and a feeling of inferiority [31]. Research supports the idea that psychological strain and stress can cause pain and somatization of symptoms [13, 14, 31].

### Methodological considerations

We addressed our topic of discussion to the SNs and not the adolescents themselves, which might represent a limitation. Equally, the SNs in our study had many years of experience with adolescents, and revealed a broad and complex understanding of adolescent pain. Focus groups as a means of data collection may be considered to have their weaknesses; for example, not everyone may get an equal time to talk. Then again, focus groups may also stimulate reflection among SNs about the topic, and thereby enrich the findings. Ideally, focus groups should not be too small to be able to facilitate a dynamic conversation between the group members. A weakness of the study is that there are few numbers of respondents in each focus group. Nevertheless, the fact that we were at five different schools ensured geographic variation. Polit & Beck (2004) [27] pointed that it is possible to conduct group interviews without complying with the formal requirements. Nevertheless, it is essential to ensure sufficient breadth and elaboration of the themes. Consequently, the researchers were particularly aware of that all participants came advocated. Our impression was that the focus group discussions reflected good dialogues, resulting in an abundant material with good variability. The strength of our study is the input from a relatively large group of SNs across a range of ages and work experience. The SNs represent schools from both rural and urban areas, and we consider them representative of SNs in Norwegian schools in general. To validate the findings, the whole research group participated in the analysis process.

### Implications for practice

To improve school nursing practice, it seems important to highlight the referral practice of adolescents experiencing everyday pain. Further, there is a need for increased knowledge about the importance of the psychosocial aspects of pain in the education program of SNs. Interdisciplinary collaboration must be taken seriously by increasingly selecting as SNs those who are not merely medical collaborators. Moreover, SNs should arrange routine follow-ups with those who have no physical causes of pain (mainly with the parents’ present).

It may appear that SNs lack the tools necessary for a systematic approach to adolescents dealing with pain. Golsater et al. [41] found that a health and lifestyle tool improved the dialogue between SNs and adolescents, allowing them to focus more easily on individual needs and to detect aspects that would otherwise not be so easily detected. SNs should also participate in and improve compulsory teaching about psychosocial health (including lifestyle, body focus, and normalization of bodily ailments) as part of the education program of adolescents.

## Conclusion

The SNs mainly associated pain with adolescents themselves or significant others having high expectations concerning bodily appearance, popularity, and school efforts. SNs also interpreted adolescent pain as a consequence of difficult relations and traumatic incidents associated with significant others, and argued that adolescent lifestyle itself could result in recurrent pain. Despite this psychosocial understanding, the SNs maintained a referral practice of medical examinations, with the result that many adolescents with pain problems become shuttlecocks in the health system. Although the SNs were also generally sceptical of the tendency toward medicalization in society, it appears that they actually help maintain this tendency. Further studies that include health-promotion strategies and family-orientated interventions to improve pain problems in adolescence are highly relevant.

## Abbreviation

SN: school nurse

## References

[1] Haraldstad K, Sørum R, Eide H, Natvig GK, Helseth S (2011). Pain in children and adolescents: prevalence, impact on daily life, and parents' perception, a school survey. Scand J Caring Sci.

[2] Roth-Isigkeit A, Thyen U, Stöven H, Schwarzenberger J, Schmucker P (2005). Pain among children and adolescents: restrictions in daily living and triggering factors. Pediatrics.

[3] Petersen S, Hägglöf BL, Bergström EI (2009). Impaired health-related quality of life in children with recurrent pain. Pediatrics.

[4] Hoftun GB, Romundstad P, Rygg M (2012). Factors associated with adolescent chronic non-specific pain, chronic multisite pain, and chronic pain with high disability: the young-HUNT study 2008. J Pain.

[5] King S, Chambers CT, Huguet A, MacNevin RC, McGrath PJ, Parker L, MacDonald AJ (2011). The epidemiology of chronic pain in children and adolescents revisited: a systematic review. Pain.

[6] Hoftun GB, Romundstad P, Rygg M (2013). Association of Parental Chronic Pain with Chronic Pain in the adolescent and young adult family linkage data from the HUNT study. JAMA Pediatr.

[7] Forgeron PA, Stinson J (2014). Fundamentals of chronic pain in children and young people. Part 1.(Report). Nurs Child Young People.

[8] Hansen D, Hansen E, Holstein B (2008). Using analgesics as tools: young women's treatment for headache. Qual Health Res.

[9] Skarstein S, Rosvold EO, Helseth S, Kvarme LG, Holager T, Smastuen MC, Lagerlov P (2014). High-frequency use of over-the-counter analgesics among adolescents: reflections of an emerging difficult life, a cross-sectional study. Scand J Caring Sci.

[10] Gorodzinsky AY, Hainsworth KR, Weisman SJ (2011). School functioning and chronic pain: a review of methods and measures. J Pediatr Psychol.

[11] Hoftun GB, Romundstad PR, Zwart JA, Rygg M (2011). Chronic idiopathic pain in adolescence--high prevalence and disability: the young HUNT study 2008. Pain.

[12] Gold JI, Mahrer NE, Yee J, Palermo TM (2009). Pain, fatigue, and health-related quality of life in children and adolescents with chronic pain. Clin J Pain.

[13] Hjern A, Alfven G, Ostberg V (2008). School stressors, psychological complaints and psychosomatic pain. Acta Paediatr.

[14] Shannon RA, Bergren MD, Matthews A (2010). Frequent visitors: somatization in school-age children and implications for school nurses. J Sch Nurs.

[15] Du Y, Knopf H, Zhuang W, Ellert U (2011). Pain perceived in a national community sample of German children and adolescents. Eur J Pain.

[16] Nygren K, Bergstrom E, Janlert U, Nygren L (2012). Parents matter--but relations to parents do not explain gender differences in self-reported health in adolescents. Scand J Caring Sci.

[17] Merlijn VP, Hunfeld JA, van der Wouden JC, Hazebroek-Kampschreur AA, Passchier J (2002). Shortening a quality of life questionnaire for adolescents with chronic pain and its psychometric qualities. Psychol Rep.

[18] Kröner-Herwig B, Gassmann J, van Gessel H, Vath N (2011). Multiple pains in children and adolescents: a risk factor analysis in a longitudinal study. J Pediatr Psychol.

[19] Kozlowska K, Rose D, Khan R, Kram S, Lane L, Collins J (2008). A conceptual model and practice framework for managing chronic pain in children and adolescents. Harv Rev Psychiatry.

[20] Gatchel RJ, Peng YB, Peters ML, Fuchs PN, Turk DC (2007). The biopsychosocial approach to chronic pain: scientific advances and future directions. Psychol Bull.

[21] Rohde G, Westergren T, Haraldstad K, Johannessen B, Høie M, Helseth S, Fegran L, Slettebø Å (2015). Teachers' Experiences of adolescents' pain in everyday life: a qualitative study. BMJ Open.

[22] Pavletic AC (2011). Connecting with frequent adolescent visitors to the school nurse through the use of intentional interviewing. J Sch Nurs.

[23] Svebak S, Jensen E, Gotestam G (2008). Some health effects of implementing school nursing in a Norwegian high school: a controlled study. J Sch Nurs.

[24] Borup IK, Andersen A, Holstein BE (2011). Re-visit to the school nurse and adolescents’ medicine use. Health Educ J.

[25] Youssef NN, Murphy TG, Langseder AL, Rosh JR (2006). Quality of life for children with functional abdominal pain: a comparison study of patients' and parents' perceptions. Pediatrics.

[26] Malterud K (2011). Kvalitative metoder i medisinsk forskning: en innføring, 3.

[27] Polit DF (2004). Nursing research: principles and methods.

[28] Lask B (1986). The high-achieving child. Postgrad Med J.

[29] Garralda ME (1996). Somatisation in children. J Child Psychol Psychiatry.

[30] Poutiainen H, Levalahti E, Hakulinen-Viitanen T, Laatikainen T (2015). Family characteristics and health behaviour as antecedents of school nurses' concerns about adolescents' health and development: a path model approach. Int J Nurs Stud.

[31] Ursin H, Eriksen HR (2010). Cognitive activation theory of stress (CATS). Neurosci Biobehav Rev.

[32] Stern J (2003). Thirty years of abdominal pain. Psychoanal Psychother.

[33] Walter S (2014). Lifestyle behaviors and illness-related factors as predictors of recurrent headache in U.S. adolescents. J Neurosci Nurs.

[34] Holtedahl R (2002). The somatization patient in the modern society. Tidsskr Nor Laegeforen.

[35] Van Houdenhove B, Luyten P (2005). Beyond dualism: the role of life stress in chronic pain. Pain.

[36] Angermeyer MC, Dietrich S (2006). Public beliefs about and attitudes towards people with mental illness: a review of population studies. Acta Psychiatr Scand.

[37] Lian OS. Den moderne Eos-myten; om medikalisering som modernitetsfenomen. Sosiologisk tidsskrift. 2006;

[38] Skog D (2005). Social interaction in virtual communities: the significance of technology. International Journal of Web Based Communities.

[39] Bond L, Carlin JB, Thomas L, Rubin K, Patton G (2001). Does bullying cause emotional problems? A prospective study of young teenagers. BMJ.

[40] Festinger L (1954). A theory of social comparison processes. Human Relations.

[41] Golsater M, Sidenvall B, Lingfors H, Enskar K (2011). Adolescents' And school nurses' perceptions of using a health and lifestyle tool in health dialogues. J Clin Nurs.

